# Maternal plasma angiotensin 1-7 concentration is related to twin pregnancy chorionicity in the third trimester of pregnancy

**DOI:** 10.3389/fendo.2023.1329025

**Published:** 2024-01-08

**Authors:** Paweł Pietruski, Katarzyna Kosińska-Kaczyńska, Agnieszka Osińska, Magdalena Zgliczyńska, Kinga Żebrowska, Katarzyna Popko, Anna Stelmaszczyk-Emmel

**Affiliations:** ^1^ Department of Obstetrics, Perinatology and Neonatology, Center of Postgraduate Medical Education, Warsaw, Poland; ^2^ Department of Laboratory Diagnostics and Clinical Immunology of Developmental Age, Medical University of Warsaw, Warsaw, Poland

**Keywords:** angiotensin II, angiotensin 1-7, twin pregnancy, monochorionic, dichorionic

## Abstract

**Introduction:**

Twin gestation is related to a higher risk of hypertensive disorders in pregnancy with possible risk stratification depending on chorionicity. It may be related to differences in plasma renin-angiotensin-aldosterone components between monochorionic and dichorionic twin pregnancies. The study aimed to analyze the plasma ANG II and ANG 1-7 concentrations in women with monochorionic and dichorionic twin gestation.

**Methods:**

A prospective observational study included 79 women between 32 and 34 weeks of gestation with twin pregnancy (31 with monochorionic gestation and 48 with dichorionic gestation). Angiotensin II and angiotensin 1-7 concentrations were measured in the collected blood samples.

**Results:**

No significant differences were observed in angiotensin II concentrations between the dichorionic and monochorionic group with significantly higher levels of angiotensin 1-7 being observed in the dichorionic group. Angiotensin 1-7 level was higher than angiotensin II in 20 women (64.5%) in the monochorionic group and in 42 women (87.5%, p=0.01) in the dichorionic group. Higher plasma concentrations of angiotensin II and lower concentrations of angiotensin 1-7 were found in 5 women with gestational hypertension and in 3 with preeclampsia compared to normotensive women.

**Discussion:**

It is the first study investigating angiotensin II and angiotensin 1-7 in twin pregnancies regarding chorionicity. Our results showed that plasma angiotensin 1-7 concentration was related to chorionicity, while plasma angiotensin II level was not. In most women with twin gestation angiotensin 1-7 concentration exceeded the concentration of angiotensin II. A switch in the relation between angiotensin II and angiotensin 1-7 was observed in hypertensive pregnant women.

## 1 Introduction

The Renin-Angiotensin-Aldosterone System (RAAS) is one of the most crucial systems in maintaining blood pressure and the hydro-electrolyte balance. During pregnancy, several changes influence the RAAS balance. The expression of the majority of its components is increased during pregnancy. Maternal plasma prorenin concentration is increased with the highest levels noted at 8-12 weeks of gestation (1). In early gestation, renin is produced and released by the ovaries and decidua. High concentrations of estrogen, produced by the placenta, increase angiotensinogen (AGT) production in the liver. That leads to an increase in plasma angiotensin II (ANG II) and aldosterone levels. ANG II exerts multidirectional biological effects through two main types of receptors: AT1R and AT2R. AT1R stimulation promotes vasoconstriction, renal sodium reabsorption, sympathetic activity, aldosterone release, angiogenesis, and cell proliferation, while AT2R stimulation inhibits cell growth, increases apoptosis, causes vasodilation and is involved in fetal tissue development (2). Although ANG II concentration is increased during pregnancy, normotensive women are resistant to its vasoconstrictive affect. It is hypothesized that progesterone decreases sensitivity to ANG II. During pregnancy, the AT1 receptors are monomeric and inactivated by reactive oxygen species and, therefore, they are insensitive to ANG II (3). In the uterine arteries, AT1R expression is similar to that in non-pregnant women, while AT2R expression is increased. Their activation contributes to the maintenance of high uteroplacental blood flow (4, 5).

The placenta contains its own renin-angiotensin systems (RAS). ANG II, which is produced by the placental RAS, binds to the AT1R and stimulates cell proliferation and migration, angiogenesis and trophoblast invasion (6, 7). The placental RAS is believed to promote placentation and regulate uteroplacental blood flow.

Angiotensin 1-7 (ANG 1-7) may be directly transformed from ANG II by a homolog of angiotensin-converting enzyme (ACE2) and from angiotensin 1-9 (ANG 1-9), which arises from ANG I. It is produced by multiple organs such as the kidney, heart, hypothalamus, and ovary. ANG 1-7 interacts with the AT1R and AT2R, but also acts as a vasodilator through the Mas receptor (MasR) (5, 8). MasR activation leads to an increased nitric oxide, kinin and prostaglandin release and, thereby, increases the antioxidant capacity of tissues and prevents endothelial dysfunction (9). ACE2 is strongly expressed in the syncytiotrophoblast in early gestation, while its expression is lower at term (5). In this location, placental ACE2 may cleave maternal circulating ANG II to form angiotensin 1-7 (ANG 1-7) (5). ACE2 levels and activity are greatly increased in the maternal circulation during pregnancy and remain high throughout gestation (10). Plasma ANG 1-7 levels are significantly increased as well (4). An increased conversion of ANG II into ANG 1-7 may also occur in the kidney in pregnancy due to an increased expression of ACE2 in this organ (11).

RAAS was not studied profoundly in twin gestation until now. Koyama et al. found significantly higher concentration of aldosterone in twin in comparison to singleton pregnancy in the second trimester, while in the third trimester of pregnancy lower aldosterone levels were observed in twin gestation in comparison to singletons. In twin pregnancies the mean plasma renin activity was higher during the second trimester and lower during the third trimester of pregnancy than in singleton gestations (12). Similar results on aldosterone levels were presented by Thomsen et al. (13). No data on ANG II or ANG 1-7 are available.

Twin gestation is related to a higher risk of hypertensive disorders in pregnancy. Laine et al. published a large study on 16,174 twin pregnancies and found the risk of PE (preeclampsia) in twins to be almost four times higher in comparison with singletons, even after adjustment for other risk factors. Further risk stratification was observed to be dependent on twin pregnancy chorionicity (14). The published literature includes reports on the relation between PE occurrence and the type of chorionicity of a twin gestation. However, the results are inconclusive. Some studies revealed that PE was more common in dichorionic gestation (15–17), while other researchers found a higher incidence of PE in monochorionic pregnancies (18) or no such relation (19–21). We hypothesized that, if PE or hypertension development during pregnancy was dependent on twin pregnancy chorionicity, there might be alterations in plasma RAAS components between monochorionic and dichorionic twin pregnancies. The aim of this study was to analyze plasma ANG II and ANG 1-7 concentrations as the two most important components of the RAAS which may influence PE development in women with monochorionic and dichorionic twin pregnancy.

## 2 Material and methods

A prospective observational study was conducted in the Department of Obstetrics, Perinatology and Neonatology, Center of Postgraduate Medical Education between May 2020 and December 2022. Women with twin gestation, who were counselled in the Outpatient Clinic or hospitalized in the Department after 31 + 6 weeks of gestation were asked to give a written informed consent to participate in the study. Gestational age was calculated based on the first day of the last menstrual period or a transfer day in assisted reproductive technique procedures, and verified by the crown-rump lengths (CRL) measured between 11 and 14 weeks on ultrasound scan. In case of inconsistency of above 5 days between the due dates calculated from the last menstrual period and ultrasound scan, gestational age was derived from the ultrasound measurement. In case of CRL discordance, the measurement from the larger twin was chosen. Chorionicity was established based on ultrasound performed in the 1st trimester. Dichorionic pregnancy was diagnosed if two gestational sacs or the lambda sign was visualized and documented, while monochorionic pregnancy was confirmed if a single gestational sac or the T sign was visualized and documented (22). All the women were counselled in the Outpatient Clinic routinely once every 4 weeks (for dichorionic pregnancies) and once every 2 weeks (for monochorionic pregnancies), including an ultrasound scan. If women delivered in the Department of Obstetrics, Perinatology and Neonatology, medical data regarding the delivery and the condition of the newborns were analyzed.

The inclusion criteria comprised age over 18 years old, live twin pregnancy, chorionicity established and documented on the 1st trimester sonographic scan, verified gestational age, pregnancy between 32 + 0 and 34 + 0 weeks of gestation. Pregnancies complicated by one or two fetal deaths, miscarriage, delivery prior to 32 weeks of gestation, twin to twin transfusion syndrome (TTTS), twin anemia-polycythemia sequence (TAPS), twin reversed arterial perfusion syndrome (TRAP) were excluded from the study. Monochorionic monoamniotic gestations were excluded from the study because the gestational age at sample collection coincided with the recommended gestational age at delivery. Women suffering from pregestational hypertension were excluded.

Gestational hypertension (GH), PE and gestational diabetes mellitus were diagnosed according to the recommendations of the Polish Society of Obstetricians and Gynecologists (23). Body mass index (BMI) was calculated by dividing the body mass by the square of the body height. Obesity was defined as BMI 30 and greater. Gestational weight gain was defined as a difference between weight at delivery and pre-gravid weight. Pre-gravid weight was self-reported, while weight at delivery was measured by the hospital staff. Discordant inter-twin birth weight was diagnosed if the difference between the larger twin birth weight and the smaller twin birth weight exceeded 25% of the larger twin birth weight.

Blood samples were collected from all the participants between 32 + 0 and 34 + 0 weeks of gestation. 10 mL samples of venous blood were collected in polystyrene tubes with tripotassium versenate (K3-EDTA). Blood samples were centrifuged for 10 min at 1500 g after 30 min following the collection. The plasma was then frozen at -80 C. After completing the study group, the plasma was defrosted and ANG II and ANG 1-7 concentrations were measured. ELISA method was used to perform the measurements. Tests were performed using standardized kits by Cloud Clone Corp., (Katy, Texas, USA). The tests were performed in accordance with the manufacturer’s instructions. Absorbance readings were performed using a UVM340 plate reader (ASYS, Biogenet), and the results were analyzed using MikroWin2000 v4 software (Mikrotek Laborsysteme GmbH, Biogenet, Overath, Germany).

The study protocol was approved by the Ethics Committee at the Center of Postgraduate Medical Education (no 35/PB/2020) and the study was conducted according to the Declaration of Helsinki. Variables are described as medians (with interquartile range) or percentages. The Mann-Whitney test and the Fisher’s exact test were used for the statistical analysis. Box and whisker plots were created to visualize the results. p-values <0.05 were considered significant. The data were analyzed using Statistica version 13.1.

## 3 Results

**Figure 1:**
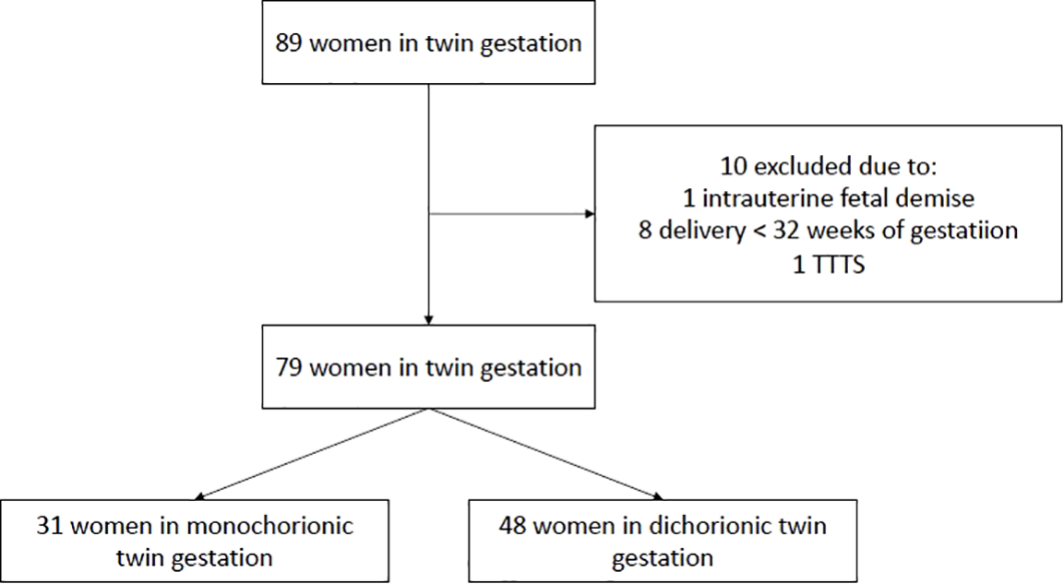
Flow chart of the study group.

79 women were recruited to the study: 31 with monochorionic gestations and 48 with dichorionic gestations. The flow chart of the study group is presented in **Figure 1**. The basic characteristics of the study group are presented in **Table 1**. No significant differences were observed between the characteristics of the dichorionic and monochorionic group.

**Table 1:** Characteristics of the study group.

	Study group n=79	Dichorionic group n=48	Monochorionic group n=31	p
	Number (%)	Number (%)	Number (%)	
Age (years)*	31 (27-37)	33 (29-38)	31 (27-36)	0.8
Primiparous	48 (60.8)	28 (58.3)	20 (64.5)	0.07
Pre-gravid BMI (kg/m2) *	63 (55-74)	64 (57-77)	62 (55-71)	0.4
Obesity	7 (8.9)	4 (8.3)	3 (9.7)	0.7
Gestational weight gain *	18 (13-25)	18 (13-27)	17 (13-24)	0.4
ART	13 (16.5)	9 (18.8)	4 (12.9)	0.5
Cohort who delivered at the Department	n=46	n=31	n=15	p
GH	5 (10.9)	3 (9.7)	2 (13.3)	1
PE	3 (6.5)	2 (6.5)	1 (6.7)	1
Early PE	1 (2.2)	1 (3.2)	0	1
Late PE	2 (4.4)	1 (3.2)	1 (6.7)	1
GDM	9 (19.6)	6 (19.4)	3 (20)	1
ICP	1 (2.2)	1 (3.2)	0	1
Gestational age at delivery (weeks) *	35 (33-36.5)	35 (32.5-36)	35 (34-37)	0.4
Cesarean section	39 (84.8)	25 (80.6)	14 (93.3)	0.4
First twin birth weight (g) *	2300 (1580-2500)	2430 (1600-2550)	2250 (1550-2500)	0.3
First twin 5th minute Apgar ≦7 points	2 (4.3)	1 (3.2)	1 (6.7)	1
Second twin birth weight (g) *	2210 (2040-2550)	2160 (2050-2620)	2230 (2010-2380)	0.4
Second twin 5th minute Apgar ≦ 7 points	3 (8.7)	1 (3.2)	2 (13.3)	0.2
Discordant inter-twin birth weight	7 (15.2)	4 (12.9)	3 (20)	0.7

*median (interquartile range).

**Figure 2:**
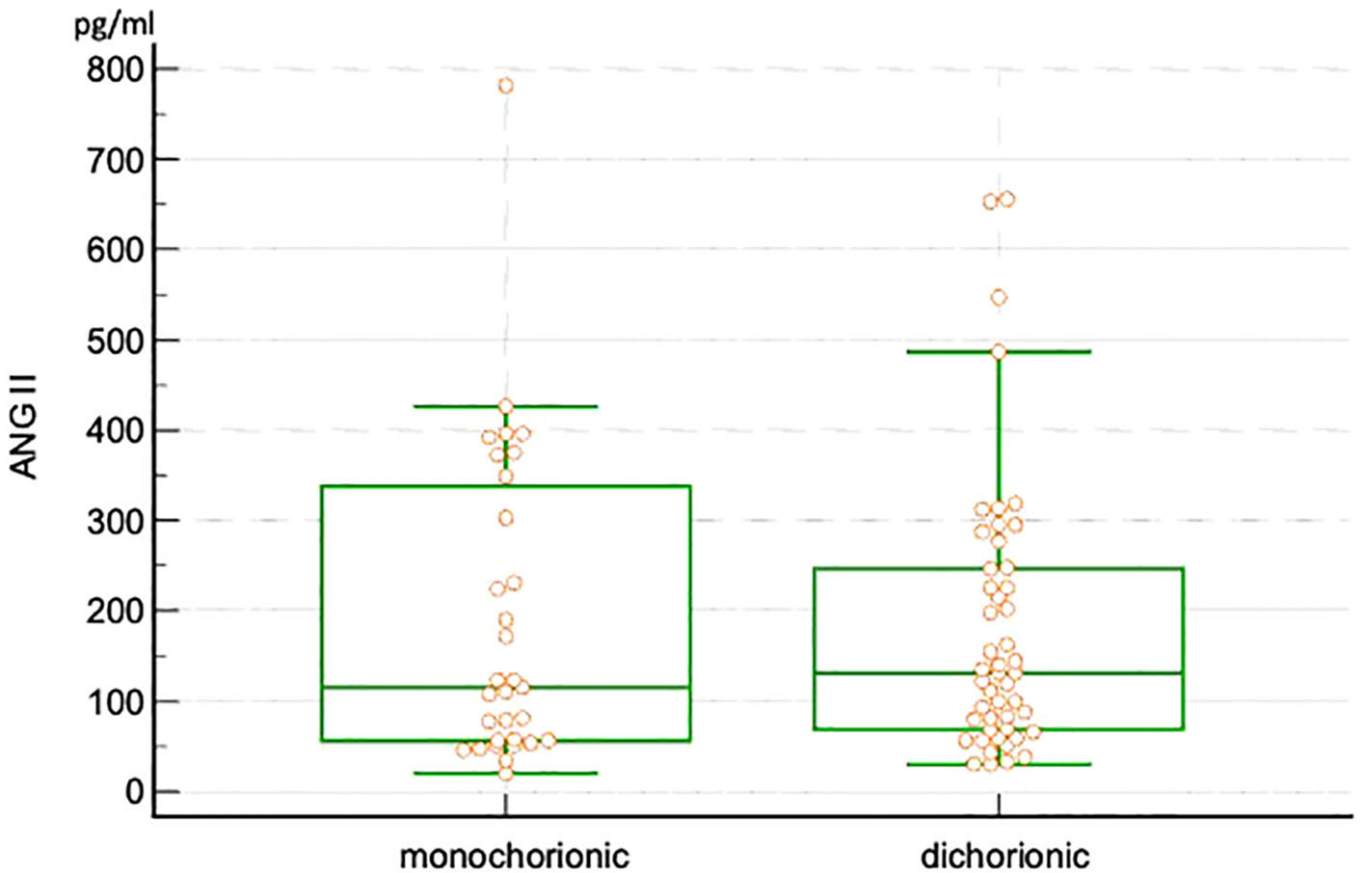
Box and whisker plots of angiotensin II in monochorionic and dichorionic twin pregnancies.

**Figure 3:**
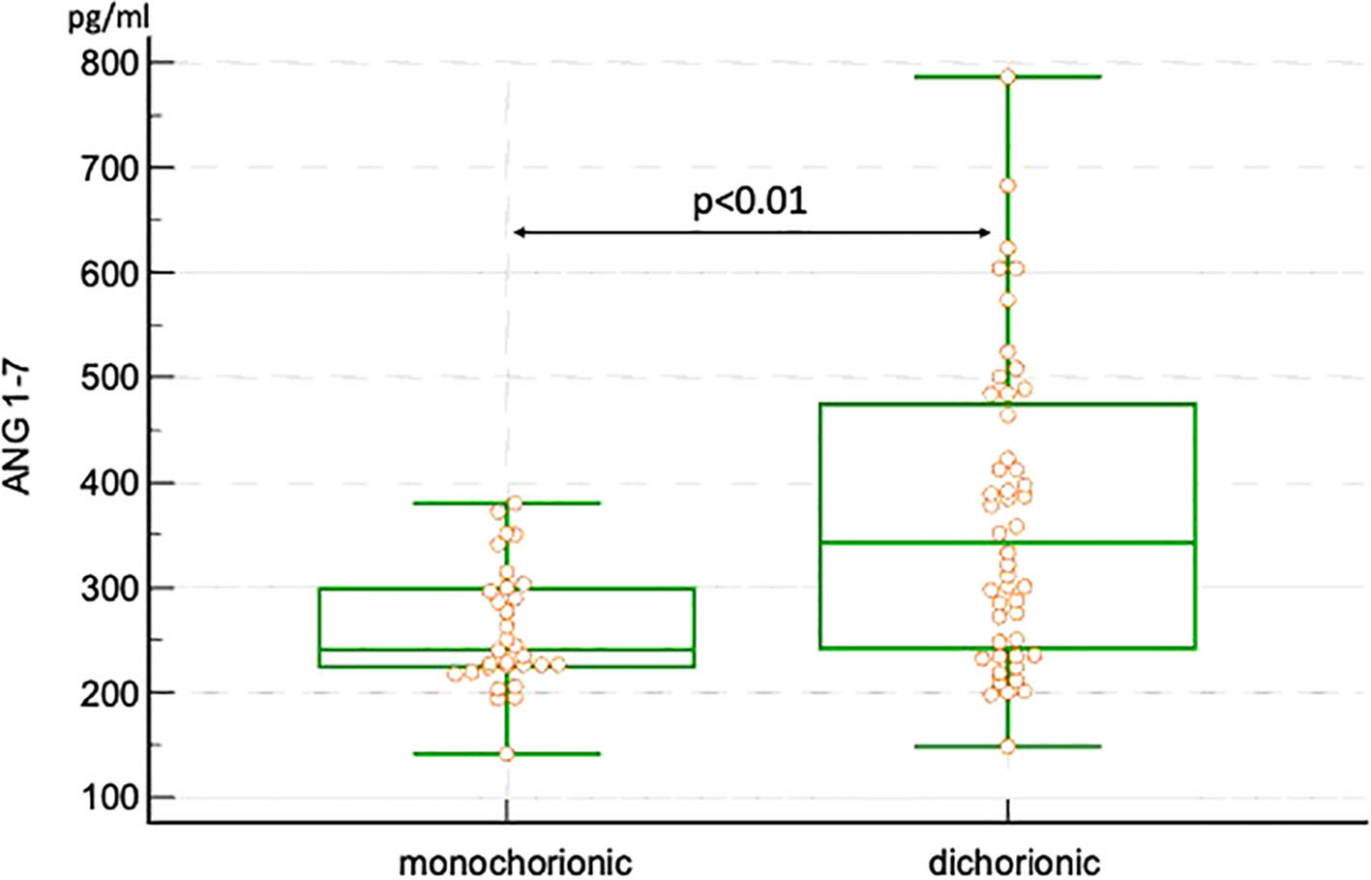
Box and whisker plots of angiotensin 1-7 in monochorionic and dichorionic twin pregnancies.

**Figure 4:**
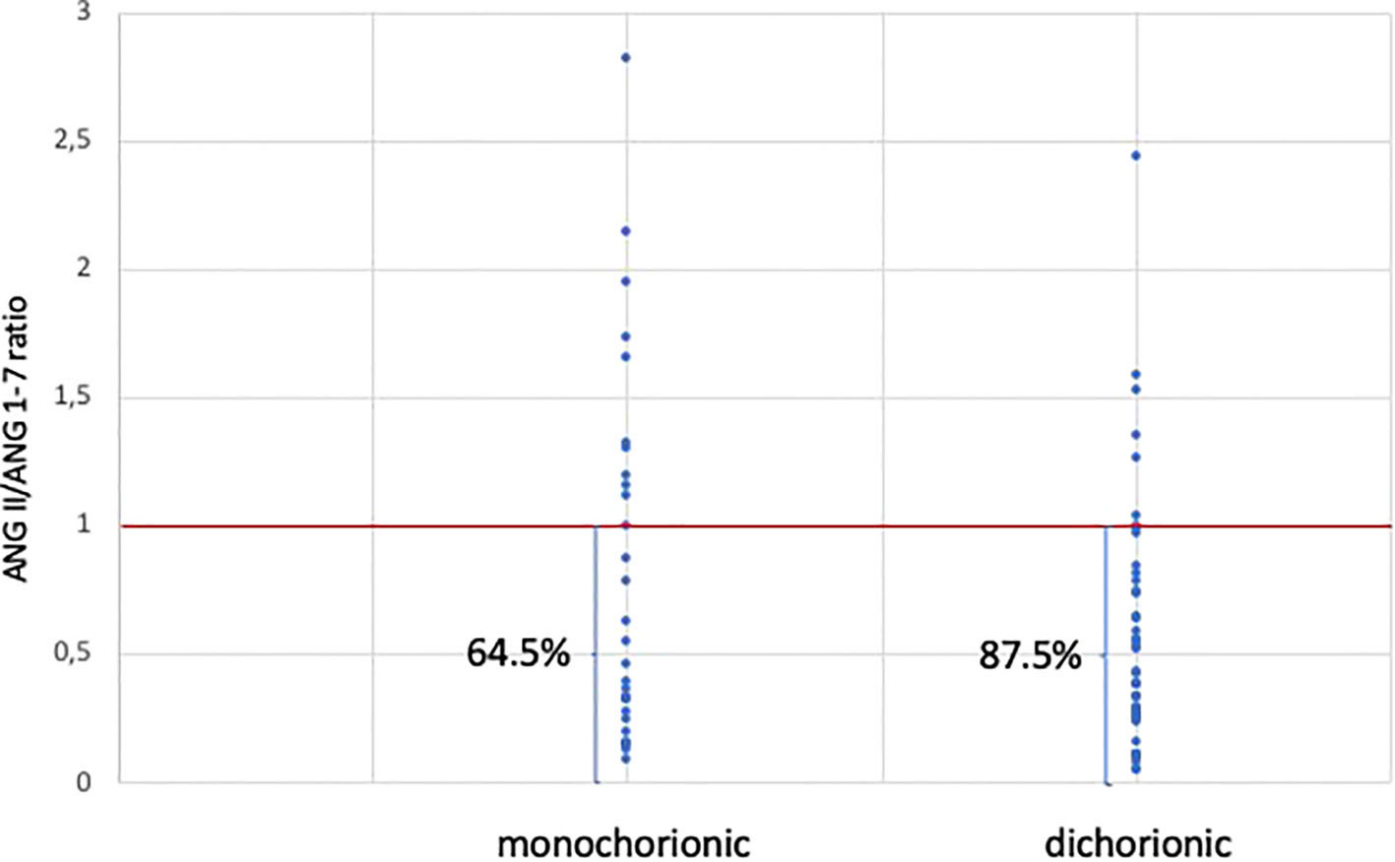
Angiotensin II to angiotensin 1-7 ratio in monochorionic and dichorionic twin pregnancies.

BMI, body mass index; ART, assisted reproductive technology; GH, gestational hypertension; PE, preeclampsia; GDM, gestational diabetes mellitus; ICP, intrahepatic cholestasis of pregnancy;

Plasma values of ANG II and ANG 1-7 are presented in **Table 2**. No significant differences were observed in ANG II levels between dichorionic and monochorionic group, while significantly higher levels of ANG 1-7 were observed in the dichorionic group. The box and whisker plots for ANG II and ANG 1-7 are presented in **Figures 2**, **3**. The ratio of ANG II to ANG 1-7 (ANG II/ANG 1-7) was calculated in both groups and did not differ significantly between the groups. In the monochorionic group, ANG 1-7 level was higher than ANG II in 20 women (64.5%), while in the dichorionic group it was higher in 42 women (87.5%, p=0.01). The ratios are presented in **Figure 4**.

**Table 2:** Serum concentrations of angiotensin II and angiotensin 1-7 in the plasma samples in the study group.

	Study group n=79	Dichorionic group n=48	Monochorionic group n=31	p
	median (interquartile range)	median (interquartile range)	median (interquartile range)	
ANG II (pg/mL)	128.25 (62.85-328.9)	131.18 (68.9-246.68)	115.33 (56.22-337.84)	0.8
ANG 1-7 (pg/mL)	312.78 (231.5-391.21)	342.54 (242.09-474.4)	240.39 (224.33-298.56)	<0.01
ANG II/ANG 1-7	0.44 (0.23-1.06)	0.38 (0.25-0.77)	0.46 (0.21-1.18)	0.2
ANG 1-7 > ANG II *	66 (83.5)	42 (87.5)	20 (64.5)	0.01

*number (%).

ANG II, angiotensin II; ANG 1-7, angiotensin 1-7; ANG II/ANG 1-7, ANG II concentration to ANG 1-7 concentration ratio; ANG 1-7 > ANG, ANG 1-7 concentration higher than ANG II concentration.

Further analysis of ANG II and 1-7 was conducted in the samples collected from women with GH and PE. The values are presented in **Table 3**. The dichorionic group included 3 pregnant women with GH and monochorionic group 2 women with GH. Although the number of cases was low, we observed that the hypertensive women had higher plasma concentrations of ANG II and lower concentrations of ANG 1-7 than normotensive patients. There were only 3 cases of PE in the study group. Two women from the dichorionic group and one from the monochorionic group developed PE. Again, the observed ANG II levels were higher than the medians of the groups, while ANG 1-7 levels lower than the group medians.

**Table 3:** Serum concentrations of angiotensin II and angiotensin 1-7 in plasma samples in women with gestation hypertension or preeclampsia.

	ANG II (pg/mL)	ANG 1-7 (pg/mL)
Dichorionic group		
Patient 1 (GH)	486.764	197.13
Patient 2 (GH)	546.508	224
Patient 3 (GH)	265.129	218.187
Patient 4 (PE)	656.152	148.319
Patient 5 (PE)	653.257	201.339
Dichorionic group (median)	131.18	342.54
Monochorionic group		
Patient 6 (GH)	375.687	141.215
Patient 7 (GH)	396.271	195.595
Patient 8 (PE)	781.495	203.509
Monochorionic group (median)	115.33	240.39

ANG II, angiotensin II; ANG 1-7, angiotensin 1-7; GH, gestational hypertension; PE, preeclampsia;

## 4 Discussion

To our knowledge this is the first study investigating ANG II and ANG 1-7 in twin pregnancies regarding chorionicity. Our results showed that plasma ANG 1-7 concentration was related to chorionicity and was significantly higher in dichorionic gestation, while plasma ANG II level was not. In most women with twin gestation, ANG 1-7 concentration exceeded the concentration of ANG II. A switch in the relation between ANG II and ANG 1-7 was observed in hypertensive pregnant women, as plasma ANG II was higher than the observed ANG 1-7 level.

In the majority of cases in our study, ANG 1-7 concentration was higher than ANG II in the maternal plasma in both groups, with the greatest difference being observed in dichorionic twin pregnancies. No results on plasma ANG II and ANG 1-7 levels in twin gestation according to chorionicity have been published to date. Merrill et al. investigated ANG I, ANG II and ANG 1-7 levels in third-trimester normotensive women with singleton gestation and found the opposite results. In most women, ANG II concentrations exceeded ANG 1-7 in the maternal plasma in their study (24). Velloso et al. investigated 20 normotensive and 20 preeclamptic women with singleton gestation in the third trimester of pregnancy. The observed mean concentration of ANG II was 66.4 ± 10.1 pg/mL in healthy singletons, while in our study the median value of plasma ANG II level was 184.1 pg/mL. The authors found the mean plasma level of ANG 1-7 in non-hypertensive singleton pregnant women to be 21.6 ± 1.1 pg/mL (25). In our group of women with twin gestation, the observed plasma values of ANG 1-7 were above 15-fold higher (median 325.3 pg/mL). We found both ANG II and ANG 1-7 levels to be higher in twin gestation compared to the data presented by Velloso et al. However, the difference was much greater for ANG 1-7.

Several changes occur in RAAS component concentration during pregnancy. On the basis of known physiological mechanisms observed in singleton pregnancy we hypothesize on the possible mechanism which may lead to higher concentrations of ANG II and ANG 1-7 in twin gestation in comparison with singletons according to the published data and the observed differences between dichorionic and monochorionic twin pregnancies. Angiotensinogen production in the liver is stimulated by estrogens. An increase in its production during singleton pregnancy is caused by estrogen secretion from the placenta. As twin placentas are bigger than those in singletons, a larger amount of trophoblast produces larger amounts of estrogens, which are released into the maternal circulation (26). Both maternal plasma estradiol and estriol concentrations were found to be significantly higher in twin versus singleton gestation by Houghton et al. (27). We hypothesize that higher estrogen levels stimulate increased angiotensinogen production in the liver. Therefore, higher amounts of angiotensinogen are released into the maternal blood in case of twin pregnancy compared to singleton gestation. As ANG II is a final product of RAAS, increased angiotensinogen production will lead to a high ANG II concentration in the maternal plasma as observed in our study. ACE2, which is present in the placenta, is responsible for the conversion of ANG II into ANG 1-7, leading to the high concentration of ANG 1-7, as observed in our study.

We observed significantly higher concentrations of ANG 1-7 in the plasma of women with dichorionic in comparison with monochorionic twin pregnancy. ACE2 plays a crucial role in ANG 1-7 production as it converts ANG II into ANG 1-7 and ANG I into ANG 1-9, which is further converted into ANG 1-7 by ACE (5). ACE activity does not change throughout pregnancy (28). ACE2, which shows approximately 40% homology with ACE, is primarily expressed in the kidney, lung, testis, heart, gastrointestinal tract, and liver endothelial cells (29). Some authors observed the upregulation of ACE2 in the endothelium and its release into the plasma in myocardial infarction (30). ACE2 is expressed in the placental syncytiotrophoblast, cytotrophoblast, and villous stroma, and it transforms ANG II into ANG 1-7. Its level is significantly increased in the maternal blood during pregnancy (9). As the placenta in dichorionic twin gestation is bigger than in monochorionic pregnancy, we hypothesize that a bigger placenta is related to a higher expression of ACE2 and, therefore, higher concentrations of ANG 1-7, as observed in our study (31). The rise in ANG 1–7 may represent conversion from ANG II by ACE2 at the placental interface.

Estrogens were found to have an impact on the RAS in animal studies. Li et al. observed a reduction in ANG II formation and augmentation in ANG 1-7 production by estrogen in a tissue-specific manner in Sprague Dawley and mRen2 transgenic rats (32). Houghton et al. investigated plasma hormone concentrations in women with singleton and twin pregnancy. The researchers found higher concentrations of maternal serum estriol in dichorionic compared to monochorionic gestations and a similar concentration of estradiol in both groups (27). As estriol is the main estrogen during pregnancy, we hypothesize that a similar mechanism of a shift in the pathways of the formation of angiotensin peptides dependent on estrogen concentration may be present in humans. The observed levels of ANG 1-7 being higher in dichorionic than monochorionic pregnancy may be related to this mechanism.

Other differences in the RAS in dependence on twin pregnancy chorionicity were observed by other researchers. The prorenin receptor contributes to the regulation of the RAS. Mikami et al. investigated maternal serum prorenin receptor concentrations as a reflection of the acceleration of tissue RAS in the placenta. The authors observed significantly higher serum prorenin receptor concentrations in dichorionic twins than in monochorionic twins. They concluded that the prorenin receptor level was related to the placenta mass and, therefore, its concentration in dichorionic twin pregnancy was higher than in monochorionic gestation (33).

Numerous authors investigated the role of the RAAS in PE development. In PE, the circulating levels of renin, ANG I, ANG II, and ANG 1-7 are decreased (3, 8, 34). Conversely, preeclamptic women demonstrated increased vascular sensitivity to ANG II (3, 8, 34). It may be related to the heterodimerization of the AT1R, which remains active in this form during pregnancy and is hyper-responsive to ANG II (8). AT1R agonistic autoantibodies (AT1-AA) were described in the circulation of preeclamptic women. They were found to bind to the AT1 receptors on a variety of cells, including the trophoblasts, and to increase factors attributed to the pathogenesis of preeclampsia (8). The AT1Rs present in the trophoblast influence the expression of soluble fms-like tyrosine kinase-1 (sFlt-1) (8). We have previously described differences in sFlt-1 levels in twin pregnancy regarding chorionicity. In a cohort study of 43 monochorionic and 36 dichorionic twin gestations, we observed significantly higher concentrations of sFlt-1 in the dichorionic group during the third trimester of pregnancy (35). It is possible that a larger trophoblast in dichorionic pregnancy produces higher amounts of sFlt-1 or due to trophoblast ischemia and hypoxia, which is related to inadequate intervillous space in a larger placenta, so more sFlt-1 is released into the maternal circulation. Mao et al. developed a human model of placental hypoxia in monochorionic twin anemia-polycythemia (TAPS) placentas. The authors found that hypoxia was associated with increased ACE2 protein levels (36). It may be hypothesized that, due to hypoxia, a higher expression of ACE2 transforms more ANG II into ANG 1-7, and ANG 1-7 concentration increases in dichorionic twin gestation.

Surprisingly higher concentrations of ANG II and lower of ANG 1-7 were found in hypertensive women in our study. As there were only few cases of GH and PE, the results may be accidental and may not reflect the entire population. However, the observed trend is very interesting, as in singletons with PE the circulating levels of ANG II, and ANG 1-7 are decreased (8). Lower ANG 1-7 levels may be related to lower activity of ACE2 on syncytiotrophoblast layer. In PE syncytiotrophoblast id hypoxic and disintegrated due to hypoxic stress and reoxygenation. It is possible that those changes may be related to lower ACE2 expression and activity. Therefore, less ANG II is transformed into ANG 1-7. Limitation of this process may be responsible for higher ANG II and lower ANG 1-7 concentrations observed in hypertensive women in twin gestation. Lower ANG 1-7 levels were found in women in singleton gestation with hypertensive disorders [(8). We hypothesize that the same mechanism is responsible for the observed effect. Higher ANG II levels may be due to bigger angiotensinogen production in liver, stimulated by estrogens, and lower transformation into ANG 1-7 by placental ACE2.

All the above presented changes in RAAS function may contribute to PE development. However, the balance between ANG II and ANG 1-7, rather than individual changes in RAAS components, seems to be crucial in the etiology of PE (37). Although only a few cases of hypertension and PE were noted in our study group, we observed a shift in ANG II/ANG 1-7 ratio between normotensive and hypertensive pregnant women.

The strength of our study is its novelty, homogeneity of the study group and a strict protocol. The time interval for blood sample collection was short (between 32 + 0 and 34 + 0 weeks of gestation) to eliminate the bias introduced by possible changes in ANG II and ANG 1-7 concentrations in relation to gestational weeks. All the laboratory assessment was performed simultaneously with the same tools and kits. The most important limitation is a small sample size. In order to verify all the hypotheses put forward in this manuscript, further research on the RAAS and uterine and placental RAS in dichorionic and monochorionic pregnancy needs to be conducted. Specific investigation of the components of the RAAS and RAS, which will explain the observed differences, will improve our understanding of placental function in twin pregnancy, as well as the etiopathogenesis of PE.

## 5 Conclusions

Maternal plasma ANG 1-7 is related to twin pregnancy chorionicity in the third trimester of pregnancy, while ANG II is not. Further studies are needed to verify hypotheses explaining the observed relation.

## Data availability statement

The raw data supporting the conclusions of this article will be made available by the authors, without undue reservation.

## Ethics statement

The studies involving humans were approved by Ethics Committee at the Center of Postgraduate Medical Education. The studies were conducted in accordance with the local legislation and institutional requirements. The participants provided their written informed consent to participate in this study.

## Author contributions

PP: Conceptualization, Data curation, Formal analysis, Methodology, Visualization, Writing – original draft. KK-K: Formal analysis, Methodology, Supervision, Writing – review & editing. AO: Data curation, Writing – original draft. MZ: Data curation, Writing – original draft. KŻ: Data curation, Writing – review & editing. KP: Formal analysis, Writing – review & editing. AS-E: Formal analysis, Writing – review & editing.
